# Adsorption of Cu(II) by Poly-γ-glutamate/Apatite Nanoparticles

**DOI:** 10.3390/polym13060962

**Published:** 2021-03-21

**Authors:** Kuo-Yu Chen, Wei-Yu Zeng

**Affiliations:** Department of Chemical and Materials Engineering, National Yunlin University of Science and Technology, Yunlin 64002, Taiwan; jackprotein@hotmail.com

**Keywords:** poly-γ-glutamate, apatite, nanoparticles, adsorption, copper ions

## Abstract

Poly-γ-glutamate/apatite (PGA-AP) nanoparticles were prepared by chemical coprecipitation method in the presence of various concentrations of poly-γ-glutamate (γ-PGA). Powder X-ray diffraction pattern and energy-dispersive spectroscopy revealed that the main crystal phase of PGA-AP was hydroxyapatite. The immobilization of γ-PGA on PGA-AP was confirmed by Fourier transform infrared spectroscopy and the relative amount of γ-PGA incorporation into PGA-AP was determined by thermal gravimetric analysis. Dynamic light scattering measurements indicated that the particle size of PGA-AP nanoparticles increased remarkably with the decrease of γ-PGA content. The adsorption of aqueous Cu(II) onto the PGA-AP nanoparticles was investigated in batch experiments with varying contact time, solution pH and temperature. Results illustrated that the adsorption of Cu(II) was very rapid during the initial adsorption period. The adsorption capacity of PGA-AP nanoparticles for Cu(II) was increased with the increase in the γ-PGA content, solution pH and temperature. At a pH of 6 and 60 °C, a higher equilibrium adsorption capacity of about 74.80 mg/g was obtained. The kinetic studies indicated that Cu(II) adsorption onto PGA-AP nanoparticles obeyed well the pseudo-second order model. The Langmuir isotherm model was fitted well to the adsorption equilibrium data. The results indicated that the adsorption behavior of PGA-AP nanoparticles for Cu(II) was mainly a monolayer chemical adsorption process. The maximum adsorption capacity of PGA-AP nanoparticles was estimated to be 78.99 mg/g.

## 1. Introduction

Heavy metal water pollution is a severe environmental issue in the developed and developing countries. Copper, one of the main heavy metal pollutants in the water, is produced from different industries like electroplating, mining, copper wire mills, coal, tanning, refining, etc. High concentration of copper is toxic to human and animals due to its high redox activity [1]. It can damage the proteins, DNA, and lipids in the cells [2]. Therefore, it is critical to explore effective and safe approaches to remove copper from polluted water.

Adsorption technique is frequently applied for removing the heavy metal pollutants from contaminated water because of its simple operation, relatively low cost, and high removal efficiency [3]. Various adsorbents are available to the removal of copper ions in wastewater, such as zeolites, clays, fly ash, apatite, activated carbons, chitosan, sodium alginate, cellulose, agricultural wastes, etc. [4]. Apatite is a calcium phosphate mineral with composition and structure similar to those of the mineral phase of natural bones and teeth. There are many types of apatite including hydroxyapatite, fluoroapatite, chloroapatite, and carbonated apatite [5]. Apatite has been utilized in wastewater treatment owing to its low water solubility, good adsorption characteristics, stable in reducing and oxidizing environments, easy preparation, facile modification, environmental-friendly, and low secondary pollution. Therefore, several studies have used apatites to remove aqueous heavy metals via ion exchange, surface complexation and dissolution/precipitation [6,7]. Nanoparticles have been studied extensively for heavy metal removal due to their high specific surface area and excellent adsorption ability [8]. There are many methods to synthesize apatite nanoparticles, such as chemical precipitation, hydrothermal treatment, emulsion method, sol-gel technique, electrodeposition, and plasma spraying process [9].

Enhanced removal of heavy metal ions through surface modification of apatite particles with carboxylic groups have been shown previously [10,11,12]. Moreover, it could increase the stability of particles in the aqueous solution. Poly-γ-glutamate (γ-PGA), a natural and biodegradable anionic polypeptide, is produced from various microorganisms such as *Bacillus subtilis* [13]. It has been widely used in medicine, cosmetics, food, agriculture, and water treatment [14]. Previous studies reported that the carboxyl and amide groups of γ-PGA can complex with various metal ions, including Cu(II), Pb(II), Hg(II), Ni(II), Mn(II), Cd(II), Fe(III), Al(III), Cr(III), and U(VI) [15,16,17]. Some researchers have synthesized apatite by wet chemical method in the presence of amino acid and polypeptide, including aspartic acid, glutamic acid poly-L-aspartate, and γ-PGA, to introduce carboxylic groups onto the apatite surface [12,18,19,20].

No investigation has been performed on Cu(II) adsorption using γ-PGA/apatite composite nanoparticles. In this study, biocompatible and environmental-friendly apatite nanoparticles were synthesized by coprecipitation method in the presence of γ-PGA to remove Cu(II) species from aqueous solution. The physical and chemical properties of the synthesized poly-γ-glutamate/apatite (denoted as PGA-AP) nanoparticles were characterized through Fourier transform infrared spectroscopy (FTIR), thermal gravimetric analysis (TGA), powder X-ray diffraction (XRD), energy-dispersive spectrometer (EDS) and dynamic light scattering (DLS). This influences of the contact time, solution pH and temperature on Cu(II) removal from aqueous solution by PGA-AP nanoparticles were studied in batch experiments. The adsorption kinetics was investigated with pseudo-first-order and pseudo-second-order models. The adsorption equilibrium was further evaluated by Langmuir and Freundlich isotherms.

## 2. Materials and Methods

### 2.1. Materials

γ-PGA with a molecular weight of about 1.2 MDa was provided by Vedan Enterprise Corporation (Taichung, Taiwan). Calcium nitrate tetrahydrate and copper nitrate trihydrate were purchased from Showa Chemical Co. (Tokyo, Japan). Diammonium phosphate, ammonia solution and nitric acid were obtained from Katayama Chemical Co. (Osaka, Japan). Sodium hydroxide was from Riedel-de Haën (Seelze, Germany). Copper standard solution (1000 mg/L) was bought from Merck (Darmstadt, Germany). All chemicals were used as received without further purification.

### 2.2. Synthesis of PGA-AP Nanoparticles

The PGA-AP nanoparticles were synthesized by coprecipitation of calcium nitrate and diammonium phosphate in the presence of γ-PGA, as reported by Boanini et al., with slight modification [21]. Briefly, 0.05 g of γ-PGA was dissolved in 50 g of deionized water and the pH of solution was adjusted to 10 with ammonia solution. Aqueous solutions of calcium nitrate and diammonium phosphate were separately prepared in concentrations of 1.08 and 0.65 M, respectively; in each was added ammonia solution to adjust pH to 10. Calcium nitrate and diammonium phosphate solutions were dropped simultaneously into the γ-PGA solution according to Ca/P molar ratio of 1.67 under magnetic stirring, and ammonia solution was added continuously to maintain the pH at 10. After dropping, the mixture was transferred to and sealed in a glass bottle, heated to 90 °C for 24 h and then cooled to room temperature. The precipitate was collected by centrifugation at 10,000 rpm for 10 min and washed three times with deionized water. Finally, the PGA-AP nanoparticles were dried at 60 °C for 24 h. The theoretical weight ratios of γ-PGA to AP were 1:20 (G1H20), 1:40 (G1H40) and 1:60 (G1H60).

### 2.3. Characterizations

The functional groups of PGA-AP were identified by a Bio-Rad FTS-40 spectrophotometer (Hercules, CA, USA) using KBr discs. A total of 64 scans within the range of 4000–500 cm^–1^ at a resolution of 2 cm^−1^ were collected and averaged for each sample.

The actual weight fraction of γ-PGA in the PGA-AP nanoparticles was roughly estimated by TGA on a TA 2050 (TA Instruments, New Castle, DE, USA). An amount of 5–10 mg of sample was heated from room temperature to 100 °C, maintained at 100 °C for 5 min and then heated to 650 °C with a heating rate of 10 °C/min. The weight fraction of γ-PGA in the nanoparticles were calculated as: (W_2_/W_1_) × 100%, where W_1_ and W_2_ represent the weight losses of γ-PGA and PGA-AP, respectively, in the 300–650 °C range [12].

The crystalline phase of PGA-AP was examined by a Rigaku MiniflexII X-ray diffractometer (Tokyo, Japan) equipped with Cu Kα radiation at 30 kV and 15 mA. Data were collected from 20° to 60° (2θ) at a scanning speed of 1°/min.

The calcium to phosphorus ratio of PGA-AP was measured by an EDS system connected to the field-emission scanning electron microscopy (JEOL JSM-6701F, Tokyo, Japan).

The particle size of PGA-AP nanoparticles was conducted by DLS (Brookhaven Instruments Co., 90Plus particle size analyzer, Holtsville, NY, USA) with deionized water as the dispersion medium.

### 2.4. Adsorption Experiments

#### 2.4.1. Procedure

The adsorption of copper ions by PGA-AP nanoparticles was performed using the batch method. An amount of 0.025 g of PGA-AP nanoparticles was contacted with 50 mL of 100 ppm of copper solutions in a shaking bath at 100 rpm. The conditions affecting copper ions adsorption onto PGA-AP nanoparticles were studied by varying pH (4, 5 and 6), temperature (30, 45 and 60 °C) and contact time (1, 3, 5, 10, 30, 60, 180 and 720 min). The pH of the solution was adjusted with nitric acid and sodium hydroxide. After adsorption experiment, the suspension was centrifuged and the supernatant was taken to determine the residual concentration of copper ions by a flame atomic absorption spectrophotometer (model AA-400, Perkin Elmer, Waltham, MA, USA). A calibration curve was obtained by measuring different concentrations of the standard copper solutions (2, 3, 4, 5 and 6 ppm) at pH values of 4, 5 and 6, which were prepared by dilutions of the stock solution (1000 mg/L) with deionized water. The amount of copper ions adsorbed by PGA-AP nanoparticles at time *t* (*q*_t_ (mg/g)) was calculated from the following mass balance equation:

(1) $$q_{t}=\frac{\left(C_{0}-C_{t}\right)V}{m}$$

where *C*_0_ and *C*_t_ (mg/L) are the concentrations of copper ions in solutions initially and at time *t*, respectively, *V* (L) is the volume of copper solution, and *m* (g) is the mass of PGA-AP nanoparticles (adsorbent). When *t* is equal to the equilibrium time, the amount of copper ions adsorbed by PGA-AP nanoparticles at equilibrium (*q*_e_) can be calculated using the above equation (i.e., *C*_t_ = *C*_e_, *q*_t_ = *q*_e_).

#### 2.4.2. Adsorption Kinetics

To understand the mechanism and kinetics of copper ions adsorption onto PGA-AP nanoparticles, the experimental data were fitted with two most common adsorption kinetic models, pseudo-first-order and pseudo-second-order model [22]. The deviation of the model from experimental data was evaluated by coefficient of determination (*R*^2^).

The pseudo-first-order model assumes that the diffusion is the rate-limiting step of the adsorption process. The rate of adsorption is proportional to the number of unoccupied sites. It can be expressed as follows:

(2) $$\frac{dq_{t}}{dt}=k_{1}\left(q_{e}-q_{t}\right)$$

The linear form is as follows:

(3) $$\ln\left(q_{e}-q_{t}\right)=\ln q_{e}-k_{1}t$$

where *k*_1_ (1/min) is pseudo-first-order rate constant. The values of *k*_1_ and *q*_e_ can be determined from the linear plot of $\ln\left(q_{e}-q_{t}\right)$ versus *t*.

The pseudo-second-order model assumes that the chemical adsorption is the rate-limiting step. The rate of adsorption is proportional to the square of the number of unoccupied sites. It can be presented as follows:

(4) $$\frac{dq_{t}}{dt}=k_{2}{\left(q_{e}-q_{t}\right)}^{2}$$

The linear form is given as:

(5) $$\frac{t}{q_{t}}=\frac{1}{k_{2}q_{e}^{2}}+\frac{t}{q_{e}}$$

where *k*_2_ (g/(mg min)) is pseudo-second-order rate constant. The values of *k*_2_ and *q*_e_ can be determined from the linear plot of *t*/*q*_t_ versus *t*.

#### 2.4.3. Adsorption Isotherms

The adsorption isotherm describes the relation between the amounts of metal ion adsorbed onto adsorbent surface and the concentration of the metal ion remaining in the solution at a particular temperature when the adsorption process reaches equilibrium conditions. Various amounts of G1H20 nanoparticles (0.025, 0.035, 0.045, 0.055, 0.065 and 0.075 g) were added to 50 mL of 100 ppm of copper solutions at pH of 6 and 60 °C. The mixture was agitated in a shaking bath at 100 rpm for 720 min. The adsorption data were fitted with two commonly used isotherm models, Langmuir and Freundlich, to understand the adsorption behavior of copper ions onto PGA-AP nanoparticles.

The Langmuir isotherm model is based on monolayer adsorption onto a homogeneous adsorbent surface without any interactions between adsorbed molecules [23]. The Langmuir equation is given by:

(6) $$q_{e}=\frac{q_{m}K_{L}C_{e}}{{1+K}_{L}C_{e}}$$

The linear form is as follows:

(7) $$\frac{C_{e}}{q_{e}}=\frac{1}{K_{L}q_{m}}+\frac{C_{e}}{q_{m}}$$

where *q*_m_ (mg/g) corresponds to the maximum amount of copper ions adsorbed per gram of adsorbent to form a complete monolayer on the surface. *K*_L_ (L/mg) is the Langmuir equilibrium constant related to the bonding-energy of the adsorbate to the adsorbent. The constants *q*_m_ and *K*_L_ can be determined from the slope and intercept of the linear plot between *C*_e_/*q*_e_ and *C*_e_.

The important characteristic of the Langmuir isotherm is the dimensionless separation factor (*R*_L_), which confirms the favorability of the adsorption process and is expressed as follows:

(8) $$R_{L}=\frac{1}{1+K_{L}C_{0}}$$

where *C*_0_ (mg/L) corresponds to the highest initial copper ion concentration in the solution.

The Freundlich isothermal model indicates the heterogeneity of the adsorbent surface and describes multilayer adsorption with an energetic nonuniform distribution [24]. The adsorption capacity is related to the concentration of copper ions at equilibrium and can be expressed as:

(9) $$q_{e}=K_{F}C_{e}^{1/n}$$

The linear form is as follows:

(10) $$\ln q_{e}=\ln K_{F}+\frac{1}{n}\ln C_{e}$$

where *K*_F_ [(mg/g)/(mg/L)^–^^1/*n*^] and 1/*n* are the Freundlich constants related to the capacity and intensity of the adsorption, respectively. The constants *K*_F_ and 1/*n* can be determined from the intercept and slope of the linear plot between ln*q*_e_ and ln*C*_e_.

## 3. Results and Discussion

### 3.1. PGA-AP Characteristics

The nucleation and growth of three PGA-AP nanoparticles in the presence of γ-PGA was performed by varying the theoretical weight ratios of γ-PGA to AP. The chemical composition and crystal structure of PGA-AP were characterized by FTIR, EDS, TGA and XRD analyses.

**Figure 1:**
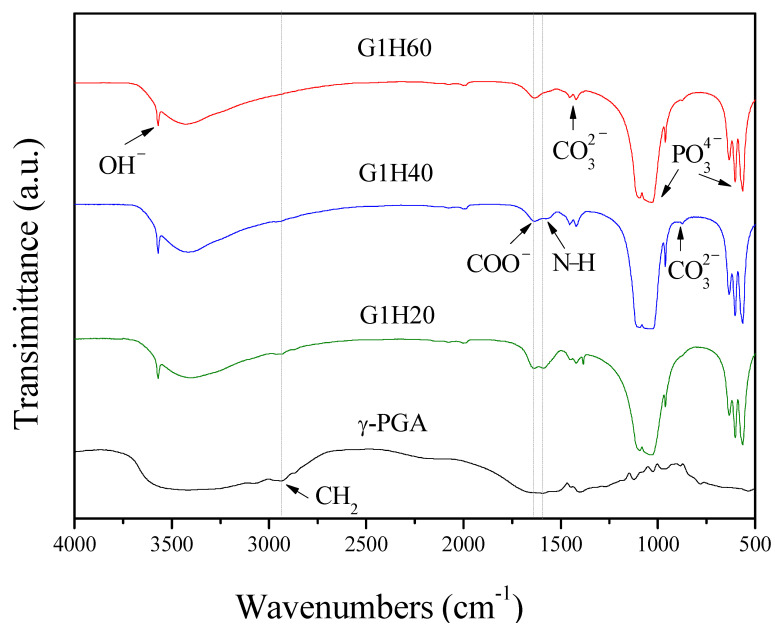
FTIR absorption spectra of poly-γ-glutamate (γ-PGA) and poly-γ-glutamate/apatite (PGA-AP) nanoparticles.

The FTIR spectrum of γ-PGA exhibited a strong and broad band around 3400 cm^–1^ (Figure 1), which could be attributed to both N–H and O–H stretching vibrations of amide groups and absorbed water, respectively [25]. The band around 2935 cm^–1^ belonged to C–H stretching vibration of alkyl chains. The N–H bending of amide II and stretching vibration of carboxylate salt group (COO^−^) appeared at around 1589 and 1635 cm^–1^, respectively [26]. The absorption band due to C=O stretching (amide I) was overlapped by that of COO^−^ [27]. In comparison of the FTIR spectra of PGA-AP and γ-PGA, PGA-AP exhibited additional strong and sharp bands at 564, 603, 634, 962, 1031 and 1095 cm^–1^ corresponding to stretching and bending vibrations of phosphate groups [28]. A sharp absorption band at 3570 cm^−1^ was due to hydroxyl ions (OH^−^) stretching vibration. The weak absorption bands at 874, 1421 and 1455 cm^–1^ attributable to CO_3_^2−^ ions, revealing that carbonate groups were incorporated into the apatite structure by substituting B-site of phosphate groups during synthesis [29,30]. The characteristic bands of γ-PGA were also shown in all the PGA-AP nanoparticles, indicating that γ-PGA was immobilized on the nanoparticles. Moreover, the relative intensities of the absorption bands at 2935, 1589 and 1635 cm^–1^ decreased with decreasing the γ-PGA content, indicating that less amount of γ-PGA was immobilized on the G1H60.

**Figure 2:**
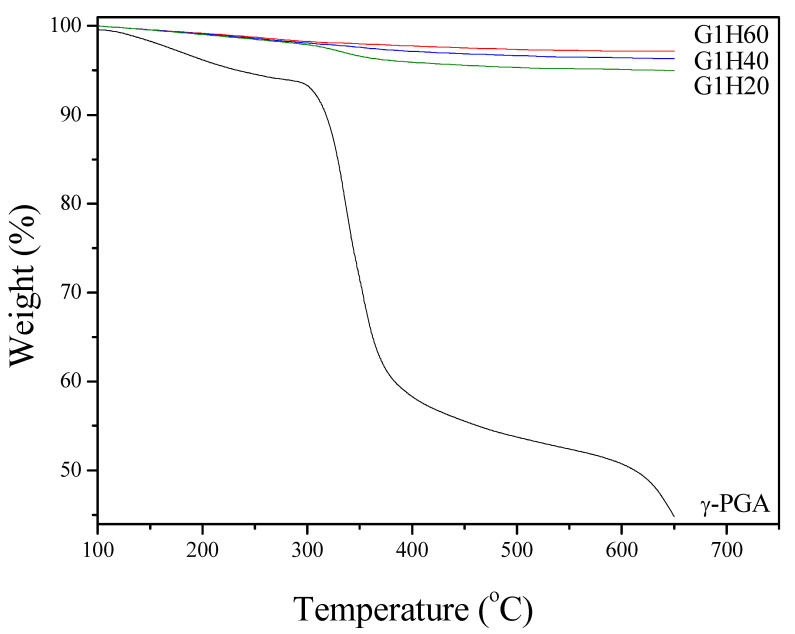
Thermal gravimetric analysis (TGA) curves of γ-PGA and PGA-AP nanoparticles.

TGA was performed to quantitatively calculate the actual weight fraction of γ-PGA in the nanoparticles. Figure 2 shows TGA curves of γ-PGA and PGA-AP nanoparticles. A considerable amount of weight loss (35%) from 300 to 400°C was noticed for γ-PGA. The smaller weight loss (<6%) observed below 250 °C was attributed to water desorption because of the hydrophilic nature of γ-PGA. All PGA-AP exhibited a similar thermal behavior. The major weight loss occurred between 300 and 400 °C due to the thermal decomposition of γ-PGA present in the particles, which increased with increasing the γ-PGA content. The relative amounts of γ-PGA incorporation into G1H20, G1H40 and G1H60 were found to be about 6.0, 3.6 and 2.2 wt%, respectively.

**Figure 3:**
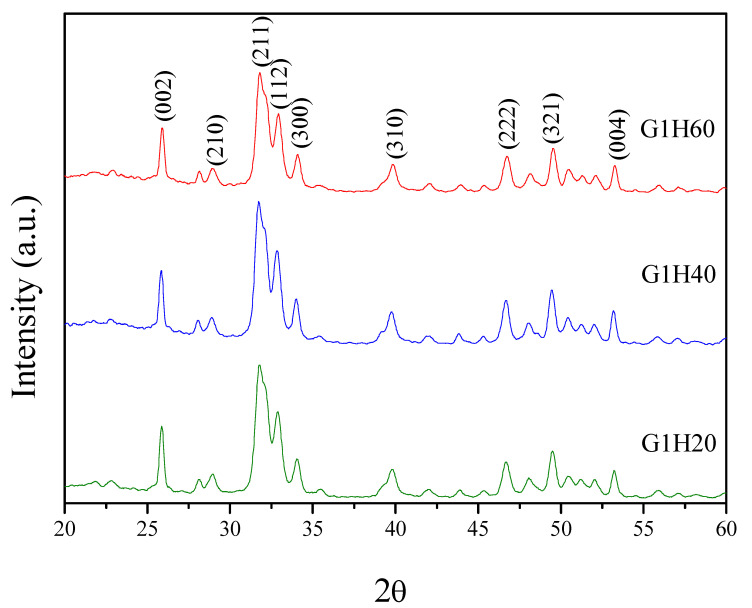
Powder X-ray diffraction (XRD) patterns of PGA-AP nanoparticles.

XRD analysis was performed to identify the crystal phase of the synthesized precipitates. The XRD patterns of PGA-AP nanoparticles are illustrated in Figure 3. PGA-AP exhibited characteristic crystalline peaks at 2θ = 25.9°, 28.9°, 31.8°, 32.9°, 34.1°, 39.8°, 46.8°, 49.5° and 53.3° corresponding to (002), (210), (211), (112), (300), (310), (222), (321) and (004) crystallographic planes, respectively, which are the major reflections of hydroxyapatite crystals [19]. Therefore, the crystallographic data indicated the formation of hydroxyapatite crystals in the presence of γ-PGA. EDS analysis showed that the Ca/P atomic ratios of G1H20, G1H40 and G1H60 nanoparticles were 1.62, 1.58 and 1.76, respectively, which were close to the stoichiometric value for hydroxyapatite (1.67).

The particle sizes of G1H20, G1H40 and G1H60 measured by DLS were 79.7 ± 1.6, 88.5 ± 0.8 and 92.8 ± 2.3 nm, respectively, indicating they increased significantly with decreasing the γ-PGA content (*P* < 0.05).

### 3.2. Adsorption Study

**Figure 4:**
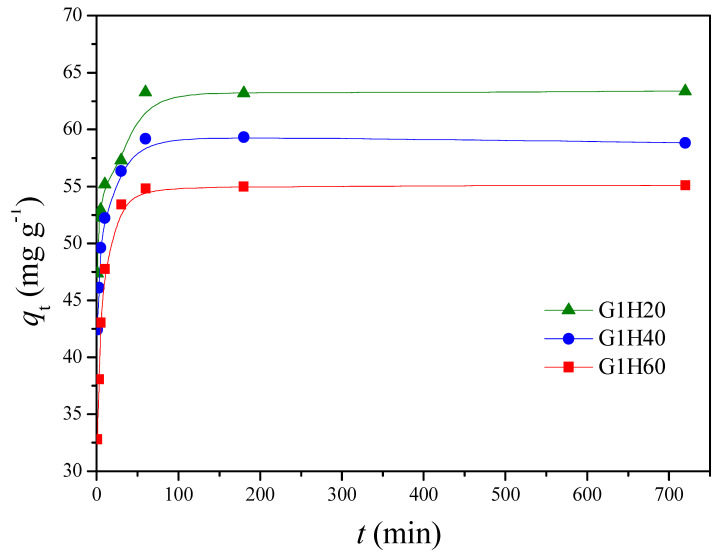
Comparison of Cu(II) adsorption onto PGA-AP nanoparticles with different weight ratios of γ-PGA to AP at a pH of 6 and 30 °C.

The adsorption experiments were carried out to investigate the influence of different parameters, such as contact time, solution pH and temperature, on the adsorption of Cu(II) in a batch system. Figure 4 presents the adsorption capacity of Cu(II) onto PGA-AP nanoparticles with different weight ratios of γ-PGA to AP at a pH of 6 and 30 °C. The rapid adsorption was observed in the first 10 min. After that, a slight decrease was occurred until equilibrium was reached at around 60 min. Several previous studies also showed that heavy metal ions can be rapidly adsorbed by hydroxyapatites [31]. Moreover, the adsorption experiments demonstrated that the equilibrium adsorption capacity, *q*_e_ (mg/g), of Cu(II) onto PGA-AP nanoparticles was increased by increasing the γ-PGA content. The surface charges of hydroxyapatite particles is positive in acid solutions due to positively charged ≡CaOH_2_^+^ and neutral ≡P–OH species on the surface [32]. Therefore, Cu(II) adsorption onto hydroxyapatite is related to other mechanisms. The main mechanisms of Cu(II) adsorption onto hydroxyapatite include ion exchange with Ca^2+^ of the hydroxyapatite, surface complexation with the P–OH groups of the hydroxyapatite, and partial dissolution of hydroxyapatite followed by the precipitation of a Cu-containing hydroxyapatite [33]. On the other hand, γ-PGA possesses abundant negatively charged carboxylates (pKa = 4.25) [34]. Moreover, the nitrogen atom of the amide group of γ-PGA can also complex with Cu(II) [17]. Therefore, the modification of hydroxyapatite with γ-PGA can provide additional active sites for Cu(II) adsorption. More carboxylate groups were available on the surface of the nanoparticles when the γ-PGA content increased. As expected, the *q*_e_ was increased from 54.99 mg/g for G1H60 to 63.29 mg/g for G1H20 (Figure 4). Similar results have been found by other investigators. Yang et al. reported that the adsorption capacity of the hydroxyapatite nanoparticles for Cu(II) was significantly enhanced through modification with humic acid, mainly because of the introduction of phenol and carboxylic groups on the surface of the nanoparticles [11]. Bachoua et al. found that the Pb(II) adsorption capacity was improved due to grafting of aspartic acid and glutamic acid onto hydroxyapatite [12]. Moreover, the higher adsorption capacity of G1H20 could be also attributed to its smaller particle size with increased surface area, which provided more adsorption sites for the removal of Cu(II). In the following experiments, the effect of temperatures and pH values on the adsorption of Cu(II) by G1H20 nanoparticles was investigated.

**Figure 5:**
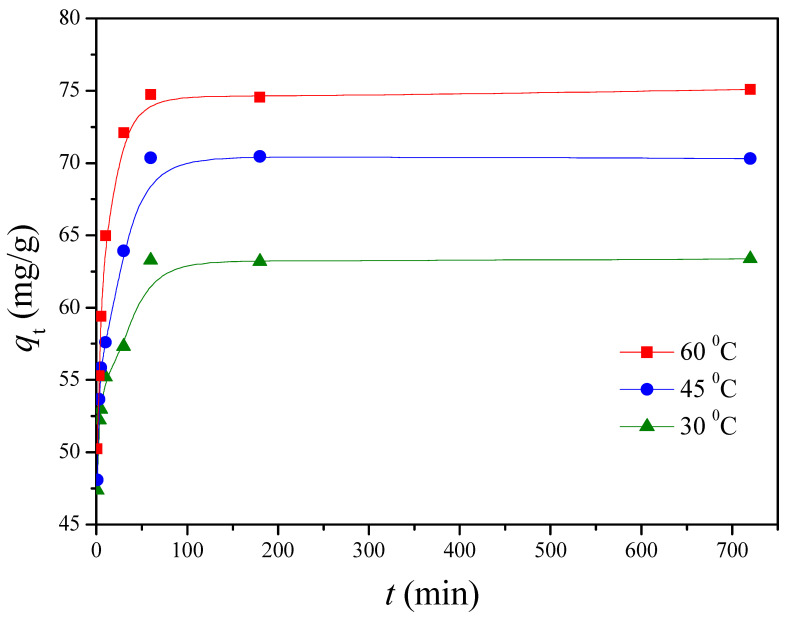
Effect of temperature on the adsorption of Cu(II) onto G1H20 nanoparticles at a pH of 6.

The effect of temperature on Cu(II) adsorption by G1H20 nanoparticles was evaluated over the range from 30 to 60 °C at a pH of 6 and 100 ppm of initial Cu(II) concentration. Figure 5 demonstrates that a rapid adsorption of Cu(II) by G1H20 was occurred within the first 10 min of contact time, followed by a gradual increase until reaching an equilibrium value. Moreover, it was observed that increasing temperature had a positive effect on the adsorption capacity of G1H20 nanoparticles. The *q*_e_ was increased from 63.29 to 74.80 mg/g as the temperature rose from 30 to 60 °C, indicating that adsorption removal of Cu(II) by PGA-AP nanoparticles favored a high temperature. Similar results have been observed by other investigators [35].

**Figure 6:**
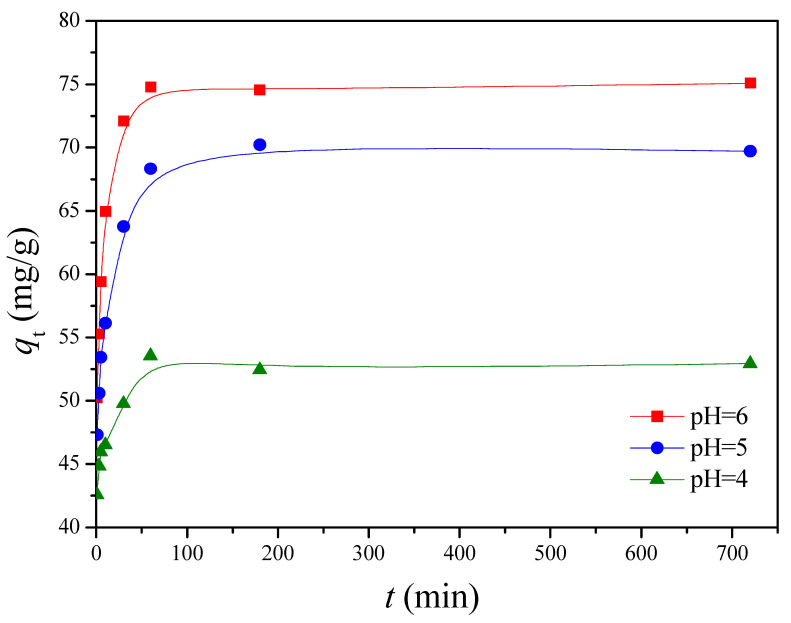
Effect of pH on the adsorption of Cu(II) onto G1H20 nanoparticles at 60 °C.

The pH of the solution is an important parameter in the removal of heavy metals from wastewater and aqueous solutions because it affects not only the degree of ionization of metal ions in solution but also the charge of adsorbents [36]. The chemical forms of Cu(II) present in aqueous solution, such as Cu(II), Cu(OH)^+^, Cu_2_(OH)_2_^2+^, Cu(OH)_2_, Cu(OH)_3_^−^, and Cu(OH)_4_^2−^, are known to depend on the pH of the aqueous solution [37]. Cu(II) is the dominant species at pH below 6.5 [38]. Solid Cu(OH)_2_ (Ksp = 2.2 × 10^–20^) starts to precipitate above pH of 6.5 [39,40]. Thus, the adsorption capability at pH beyond 6 was not studied. The influence of pH on Cu(II) adsorption by G1H20 nanoparticles was studied by varying the pH from 4 to 6 at 60 °C and 100 ppm of initial Cu(II) concentration. As shown in Figure 6, the adsorption rate of Cu(II) was rapid during the first 10 min and reached equilibrium after 60 min. The *q*_e_ was increased from 52.98 to 74.80 mg/g as solution pH increased from 4 to 6. The carboxylate groups of the G1H20 nanoparticles were partially protonated to generate carboxylic acid groups at lower pH, which results in competition between Cu(II) and hydrogen ions for the same adsorption sites [41]. Yang et al. found that the adsorbed amount of Cu(II) was increased with increasing the solution pH from 2.5 to 6.5 [11]. Siao et al. revealed that the maximum binding amounts of Pb(II) and Cd(II) by γ-PGA were occurred in the pH range of 5 to 7 [42]. They also reported that an appreciable adsorption of Hg(II) by γ-PGA was observed at pH beyond 3 and reached a maximum at a pH of 6 [15]. Additionally, the surface of hydroxyapatite particles is net positively charged at low pH conditions, which is less favorable in complexing metal cations on the adsorbent surface [43,44]. Therefore, the G1H20 nanoparticles can adsorb more Cu(II) at a pH of 6.

#### 3.2.1. Adsorption Kinetics

**Figure 7:**
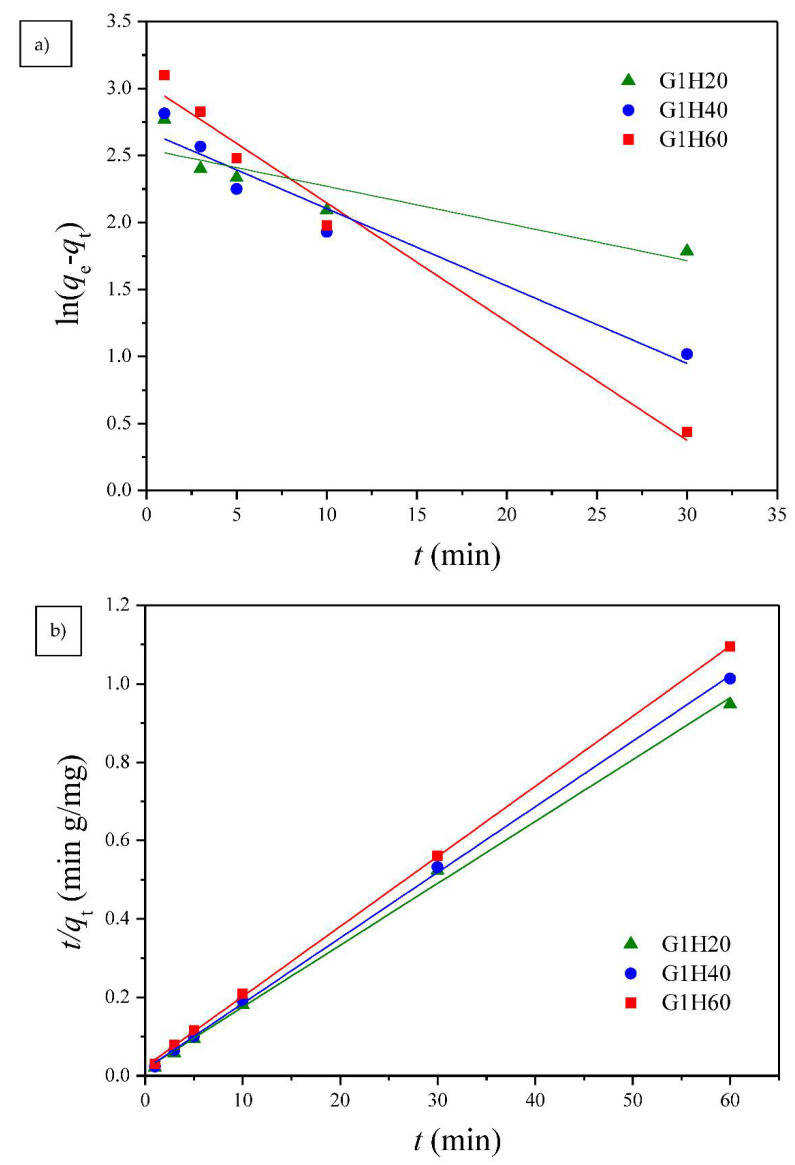
The (**a**) pseudo-first order and (**b**) pseudo-second order kinetic models for Cu(II) adsorption onto PGA-AP nanoparticles with different weight ratios of γ-PGA to AP at a pH of 6 and 30 °C.

**Figure 8:**
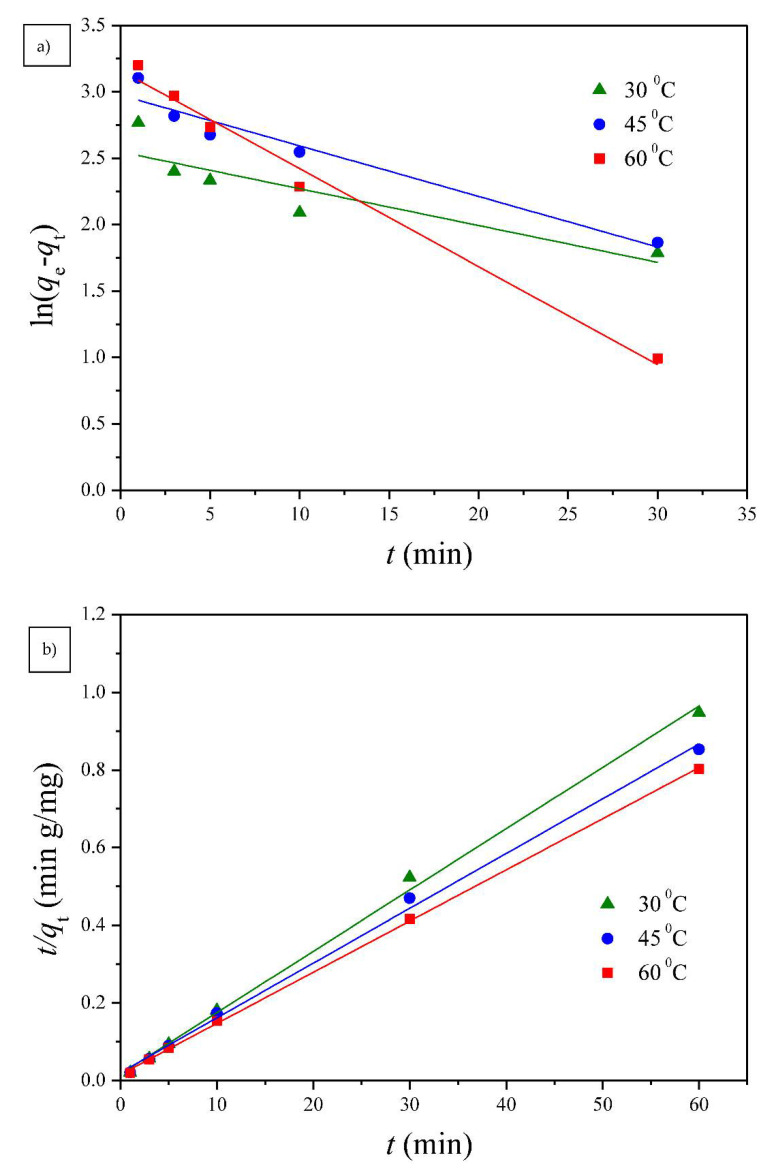
The (**a**) pseudo-first order and (**b**) pseudo-second order kinetic models for Cu(II) adsorption onto G1H20 nanoparticles at a pH of 6 and different temperatures.

**Figure 9:**
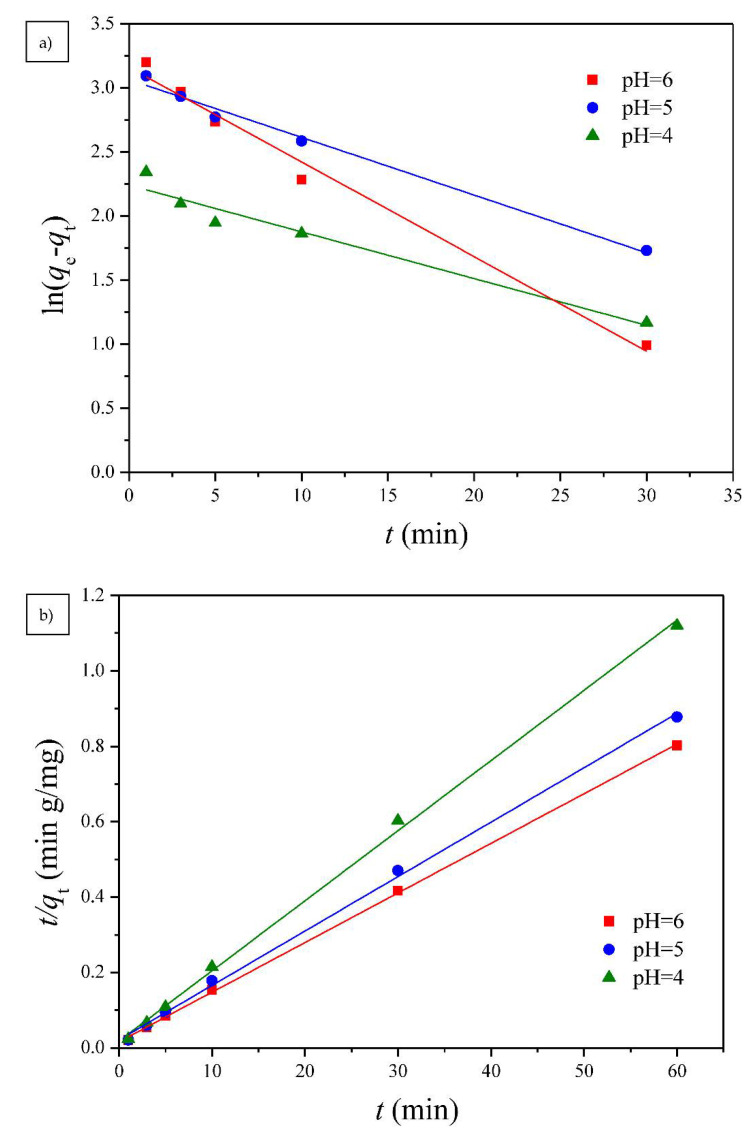
The (**a**) pseudo-first order and (**b**) pseudo-second order kinetic models for Cu(II) adsorption onto G1H20 nanoparticles at 60 °C and different pH values.

Two well-known kinetic models, a pseudo-first order equation of Lagergren based on solid capacity and a pseudo-second order equation based on solid phase adsorption, were applied to evaluate the mechanism of adsorption [43]. Figure 7, Figure 8 and Figure 9 show the linear plots of pseudo-first-order and pseudo-second-order models for the adsorption of Cu(II) onto different PGA-AP nanoparticles at different temperatures and pH values, respectively. The adsorption kinetic constants and correlation coefficients (*R*^2^) for both pseudo-first and pseudo-second-order kinetics are presented in Table 1. As shown in Table 1, The calculated *q*_e_ values from the pseudo-first-order kinetic model were much different from the experimental ones, indicating that it was not appropriate to describe the adsorption process. It is clear from *R*^2^ values that the adsorption process of Cu(II) onto PGA-AP nanoparticles obeyed the pseudo-second-order kinetic model better than the first-order kinetic model. Most of previous studies reported that the adsorption kinetics of Cu(II) by the hydroxyapatite-containing composites followed the pseudo-second-order kinetic model [45]. The *R*^2^ values for the pseudo-second-order kinetic model were nearly equal to 1 (>0.997), and the calculated *q*_e_ values agreed very well with the experimental ones. These results suggest that the rate-limiting step in the adsorption process may be chemical adsorption involving valence forces via electrons sharing or exchange between the PGA-AP nanoparticles and Cu(II) [46,47].

#### 3.2.2. Adsorption Isotherms

**Figure 10:**
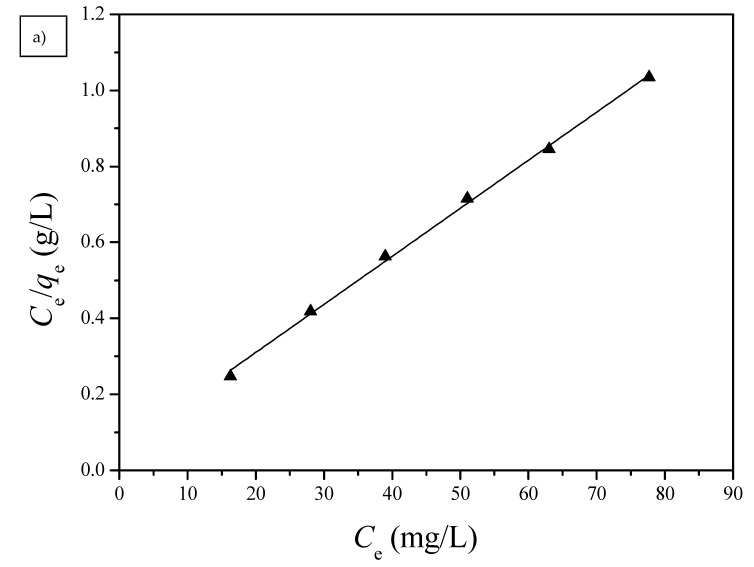

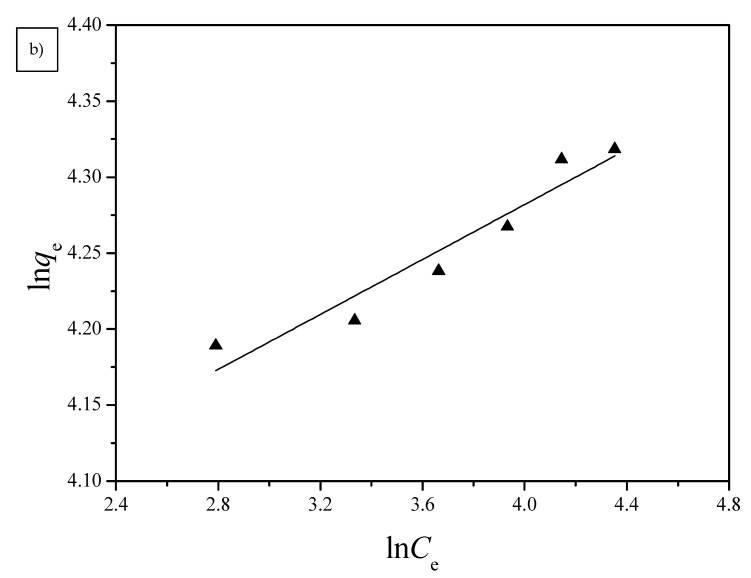
(**a**) Langmuir and (**b**) Freundlich isotherms for the adsorption of Cu(II) by G1H20 nanoparticles at a pH of 6 and 60 °C.

Langmuir and Freundlich isotherm models were used to investigate the adsorption equilibrium of the PGA-AP nanoparticles for Cu(II) adsorption. The absorption data were fitted to the linear forms of the Langmuir and Freundlich isotherms for Cu(II) adsorption onto G1H20 nanoparticles at a pH of 6 and 60 °C and are illustrated in Figure 10. The obtained isotherm model constants and *R*^2^ values are summarized in Table 2. The maximum adsorption capacity (*q*_m_) calculated from Langmuir equation (78.99 mg/g) was close to the experimental value (74.80 mg/g). The Langmuir equilibrium constant (*K*_L_) was 2.23 × 10^−1^ L/mg. The dimensionless separation factor (*R*_L_) was 0.0429, which was in the range of 0 to 1, indicating favorable adsorption of Cu(II) onto PGA-AP nanoparticles. The Freundlich adsorption capacity (*K*_F_) was determined to be 50.41. The adsorption intensity (1/*n*) was 0.0904, which was less than 1, indicating that Cu(II) was easily adsorbed by PGA-AP nanoparticles. The *R*^2^ value of the Langmuir isotherm (0.9978) is higher than the obtained value from the Freundlich isotherm (0.9066), suggesting that the adsorption of Cu(II) form a monolayer onto the surface of PGA-AP nanoparticles. The result is consistent with other previous studies [5,45,48].

## 4. Conclusions

PGA-AP nanoparticles with 2.2 to 6.0 wt% of γ-PGA were synthesized as adsorbents for removing Cu(II) from aqueous solution. The composition and structure of PGA-AP were confirmed by FTIR, TGA, XRD and EDS. The particle size of PGA-AP nanoparticles decreased significantly with the increase of γ-PGA content. It was also found that the Cu(II) adsorption amount of PGA-AP nanoparticles was increased with increasing the immobilized amount of γ-PGA. The equilibrium adsorption capacity of about 74.80 mg/g was achieved at pH of 6 and 60 °C after 60 min of contact time. The adsorption kinetics and isotherms investigations demonstrated that the Cu(II) adsorption by PGA-AP nanoparticles followed well the pseudo-second-order kinetics and the Langmuir isotherm equation, respectively. It suggests that the Cu(II) adsorption process was mainly chemical adsorption and monolayer adsorption.

## Figures and Tables

**Table 1:** Pseudo-first-order and pseudo-second-order kinetic model constants for Cu(II) adsorption onto PGA-AP nanoparticles.

Materials	Temperature	pH	*q*_e_, exp (mg/g)	Pseudo-First-Order Model Constants			Pseudo-Second-Order Model Constants		
	(°C)			*q*_e_, cal (mg/g)	*k*_1_ (1/min)	*R* ^2^	*q*_e_, cal (mg/g)	*k*_2_ (g/(mg min))	*R* ^2^
G1H20	30	6	63.29	12.79	2.78 × 10^−2^	0.7303	63.37	1.42 × 10^−2^	0.9971
G1H40	30	6	59.13	14.62	5.78 × 10^−2^	0.9348	59.77	1.63 × 10^−2^	0.9993
G1H60	30	6	54.99	20.77	8.86 × 10^−2^	0.9783	55.96	1.35 × 10^−2^	0.9997
G1H20	45	6	70.37	19.60	3.81 × 10^−2^	0.9298	70.92	9.52 × 10^−3^	0.9971
G1H20	60	4	52.98	9.42	3.65 × 10^−2^	0.9416	53.73	1.90 × 10^−2^	0.9983
G1H20	60	5	69.41	21.45	4.51 × 10^−2^	0.9866	69.20	9.93 × 10^−3^	0.9983
G1H20	60	6	74.80	23.62	7.39 × 10^−2^	0.9834	75.93	1.09 × 10^−2^	0.9995

**Table 2:** Langmuir and Freundlich isotherm constants for Cu(II) adsorption onto G1H20 nanoparticles at a pH of 6 and 60 °C.

Langmuir Isotherm Model				Freundlich Isotherm Model		
*q*_m_ (mg/g)	*K*_L_ (L/mg)	*R*_L_	*R* ^2^	*K*_F_ [(mg/g)/(mg/L)^–1^*^/n^*]	1/*n*	*R* ^2^
78.99	2.23 × 10^−1^	4.29 × 10^−2^	0.9978	50.41	9.04 × 10^−2^	0.9066

## Data Availability

Data is contained within the article.
